# Association of Vestibular Neuritis Following COVID-19 Vaccination

**DOI:** 10.15190/d.2024.14

**Published:** 2024-09-30

**Authors:** Selia Chowdhury, Nurjahan Shipa Chowdhury

**Affiliations:** ^1^Jersey Shore University Medical Center, Neptune City, New Jersey, USA; ^2^Dinajpur Medical College, Dhaka, Bangladesh.3 Medical College Baroda, Vadodara, India

**Keywords:** Covid-19, vaccination, vestibular neuritis, vertigo.

## Abstract

Among the various side-effects of coronavirus disease 2019 (COVID-19) vaccinations, vestibular neuritis (VN) has been found to have some interesting association with the vaccinations. This paper mainly focuses on exploring different associations between COVID-19 vaccination and VN. A systematic search was conducted on electronic databases including PubMed, Google Scholar, and Cochrane using MeSH terms for case reports published until July 2023. A total of 6 case reports involving 7 individuals from 6 different countries were documented. Reports were analyzed to identify presenting symptoms, diagnosis, treatment, and pathophysiological mechanisms related to the relevant issues. The studies included a diverse range of individuals with ages ranging from 40 to 61 years, with an average age of 51 years and a male predominance. The average time between vaccination and symptom onset was 6.35 days. Prominent clinical features observed in the case reports included acute onset vertigo, nausea, vomiting, nystagmus, and gait instability. Diagnostic studies primarily involved vestibular test and brain imaging. Available treatment options consisted of vestibular suppressants, steroids and vestibular rehabilitation. This review highlights the diverse and clinically relevant associations between COVID-19 vaccination and vestibular neuritis. The findings underscore the importance of conducting further studies to explore the causative links in this correlation and gain a better understanding of the relationship.

## INTRODUCTION

The coronavirus disease 2019 (COVID-19) pandemic, caused by the severe acute respiratory syndrome coronavirus 2 (SARS-CoV-2), has highlighted the urgency and significance of COVID-19 vaccination on a global scale^1^. Vaccination against COVID-19 is crucial in mitigating the spread of the disease and reducing its severe manifestations, including pneumonia^2^. However, the occurrence of uncommon vaccine-related adverse events, some with the potential for fatality, has raised concerns. Notably, various neurological complications have been reported in association with COVID-19 vaccination, including Guillain-Barré syndrome (GBS), encephalitis, seizures, acute stroke, delirium, meningoencephalitis, and facial nerve palsy^3^. Facial nerve palsy has been attributed to the reactivation of the herpes simplex virus (HSV) or varicella-zoster virus (VZV)^4^, and instances of VZV reactivation following COVID-19 vaccination have been documented^5^. Additionally, several reports in the fields of dermatology and ophthalmology have indicated diseases associated with VZV reactivation subsequent to COVID-19 vaccination^6^. While vestibular neuritis (VN) is considered to be linked to HSV reactivation^7^, reported cases associating VN with COVID-19 vaccination have been scarce. Vestibular neuritis is the third most prevalent peripheral vestibular disease, after benign paroxysmal positional vertigo and Ménière disease. It is characterized by sudden spontaneous vertigo without hearing loss^8^. It falls under the category of acute vestibular syndrome, along with multiple sclerosis, and stroke^9^. Although viral inflammation or reactivation of latent viruses in the ganglion of the vestibular nerve is believed to trigger vestibular neuritis^8^, the exact etiology remains unclear. In light of these observations, this systematic review aims to conduct a comprehensive analysis of published case reports to assess the possibility of vestibular neuritis occurrence after COVID-19 vaccination. disease. To our knowledge, this is the first systematic review to discuss such association. Specifically, this study seeks to explore various associations between COVID-19 vaccination and vestibular neuritis, shedding light on potential causal relationships and contributing to the existing knowledge in this area.

## METHODS

**Figure 1:**
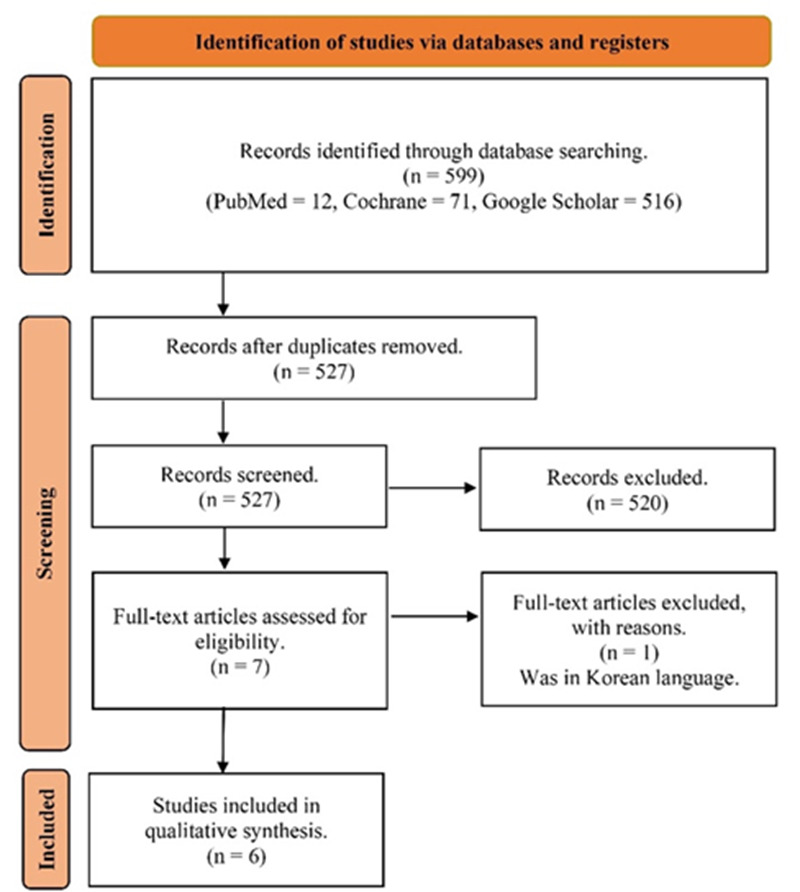
PRISMA flow diagram showing selection process of the studies included in this review.

A comprehensive search was conducted on PubMed, Google Scholar, and Cochrane databases to identify relevant case reports published up until July 2023. The search terms used were "vestibular neuritis" or "VN", “acute unilateral peripheral vestibulopathy” or “AUPV”, "COVID-19 vaccines" or "COVID-19", "vaccination" or "vaccine", and "case report." Initially, the screening process involved reviewing the titles and abstracts of the articles. Subsequently, 6 case reports that focused on presenting symptoms, pathophysiological mechanisms, diagnosis, and treatment were considered (Figure 1).

## RESULTS

**Figure 2:**
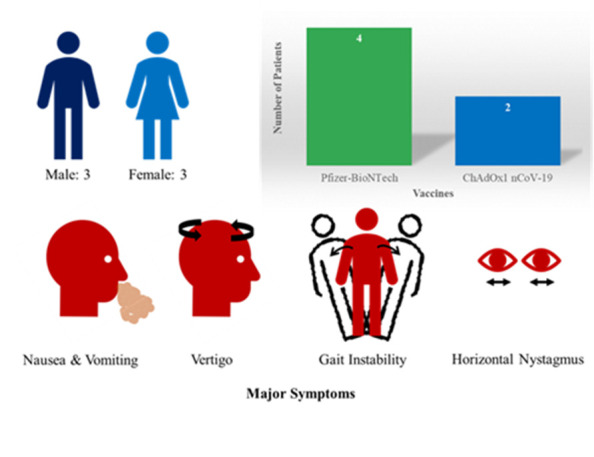
Infographic showing patient demographic, vaccine frequency, and major symptoms

In our study, we analyzed 6 case reports published in peer-reviewed journals. These reports involved a total of 6 individuals who developed VN following COVID-19 vaccination. Full texts from the case reports were reviewed, and extraction of relevant data was then conducted and organized into tables (Table 1 and Table 2). Among the cases reviewed, the highest number of VN cases, 4 individuals, were reported after receiving the Pfizer-BioNTech vaccine. This was followed by 2 cases after ChAdOx1 nCoV-19 (AstraZeneca) vaccination, as depicted in Figure 2. Most of the patients developed VN following the first dose of the vaccine (n=5, 83.33%), and one case after the booster dose.

The incidence of VN development following COVID-19 vaccination was reported in case reports from six different countries, one case each from the USA, UK, Switzerland, South Korea, Iran, and Mexico. The individuals affected had an age range of 40 to 61 years. The average age at which VN occurred after receiving the respective COVID-19 vaccine was determined to be 49.33 years.

**Table 1:** Patient Characteristics of included studies

	Study	Publication Date	Country	Age (Years)	Sex	Comorbidity	Vaccine Type	Dose
1	Jeong et al.^10^	12/13/2021	South Korea	54	Male	Hypertension	Pfizer-BioNTech	First
2	Medina et al.^11^	1/3/2022	Mexico	40	Female	None	ChAdOx1 nCoV-19	First
3	Aliakbar et al.^12^	5/2/2022	USA	40	Male	Eczema Arthritis	Pfizer-BioNTech	Third
4	Ekobena et al.^13^	5/5/2022	Switzerland	61	Female	Hypertension Atrial Fibrillation	Pfizer-BioNTech	First
5	Bramer et al.^14^	6/6/2022	United Kingdom	50	Female	-	Pfizer-BioNTech	First
6	Shahali et al.^15^	7/25/2022	Iran	51	Male	None	ChAdOx1 nCoV-19	First

No significant gender difference was observed, as there were an equal number of males (3 out of 6 individuals) and females (3 out of 6 individuals) in the study sample. The duration between receiving the COVID-19 vaccine and the onset of VN symptoms (latency period) ranged from 12 hours to 21 days, the average duration was recorded as 7.33 days. The patients in this study have comorbidities that include hypertension, arthritis, eczema, and atrial fibrillation.

The clinical presentations of VN included acute, severe vertigo (n = 5, 83.33%), vomiting (n = 4, 66.67%), nausea (n = 4, 66.67%), horizontal nystagmus (n = 4, 66.67%), gait instability (n = 2, 33.33%), inability to walk, etc. There were no documented reports of myalgia or fever or any systemic symptoms in the cases reviewed. Additionally, there were no serologic findings suggestive of inflammation. The diagnosis of VN relies on clinical history, examination, vestibular tests (caloric test, head impulse test etc.), and supplementary investigations such as magnetic resonance imaging (MRI) of the brain and internal auditory canal, pure-tone audiometry (PTA), and cerebral computed tomography (CT) arteriography. Decreased vestibulo-ocular reflex gain was found in head impulse test (n=4). Due to the severity of symptoms, no vestibular testing could be obtained in one case^11^. MRI of the brain was done on three patients and was inconclusive.

There are several therapies for vestibular neuritis, which may be broadly classified as symptomatic therapy, specialized medication therapy, and vestibular rehabilitation therapy. Nausea and vomiting were prevalent during the acute stage of vestibular neuritis. Hence, in situations where consuming food was challenging, it was necessary to provide adequate fluid infusion along with the administration of vestibular suppressants and antiemetics. Vestibular suppressants are drugs that reduce the intensity of vertigo and nystagmus evoked by a vestibular imbalance. Vestibular suppressants consisted of muscarinic antagonist (diphenidol), histamine analogue (betahistine), and benzodiazepine (lorazepam). Corticosteroids were administered in 3 patients (50%). 4 patients needed vestibular rehabilitation. The clinical symptoms improved in 2 to 10 days and the patients returned to their baseline (n=4, 66.67%). Two of the patients had partial recovery despite proper treatment and rehabilitation.

## DISCUSSION

This systematic review aimed to assess the association between COVID-19 vaccination and VN by analyzing published case reports. Our findings revealed several cases of VN occurring after COVID-19 vaccination, indicating a potential link between the two. The reported cases of VN following COVID-19 vaccination highlight the importance of monitoring and investigating potential adverse events associated with vaccines. While the overall number of cases was relatively small, the occurrence of VN post-vaccination raises concerns about the safety profile of COVID-19 vaccines. It is crucial to emphasize that these cases represent a rare occurrence, and the benefits of COVID-19 vaccination in preventing severe illness and reducing transmission far outweigh the risks of adverse events.

**Table 2:** Clinical presentation and outcome of vestibular neuritis patients following COVID-19 vaccination * IV: Intravenous, MRI: Magnetic Resonance Imaging, PTA: Pure Tone Audiometry, CT: Computed Tomography.

SN	Latency	Clinical Presentation	Diagnosis	Management	Outcome
1	3 days	· Acute vertigo · Right horizontal-torsional spontaneous nystagmus	· Caloric test: Normal · Video head impulse test: Decreased vestibulo-ocular reflex gain · Cervical vestibular evoked myogenic potential: Responses on both sides · Dynamic posturography: Low composite score	· Vestibular suppressants · Vestibular rehabilitation	Full recovery
2	21 days	· Severe vertigo · Nausea, vomiting. · Inability to walk. · Grade III horizontal nystagmus	· Due to the severity of symptoms, no vestibular testing could be obtained.	· IV diphenidol, ondansetron, and dexamethasone	Full recovery
3	1 day	· Nausea, lightheadedness, and poor coordination · Truncal ataxia · Broad base slow gait	· MRI brain was negative and nonrevealing	· Meclizine and Zofran	Full recovery
4	5 days	· Vertigo with gait instability · Nausea, vomiting. · moderate headache · Spontaneous horizontal nystagmus	· Positive head impulse test · Decreased vestibulo-ocular reflex gain · Brain MRI was normal	· Antiviral · Prednisone · Candesartan · Vestibular physiotherapy	Partial recovery
5	3 days	· Acute vertigo · Vomiting · Horizontal nystagmus	· Impaired vestibulo-ocular reflex	· Prochlorperazine · Betahistine · Vestibular rehabilitation for 6 weeks	Full recovery
6	11 days	· Severe constant true-whirling vertigo · Nausea, vomiting	· Abnormal caloric test · Positive bedside head impulse tests · PTA, MRI of the brain and internal auditory canal, and cerebral CT arteriography were normal.	· Methylprednisolone · Antiemetic · Lorazepam · Vestibular rehabilitation	Partial Recovery

The underlying mechanisms linking COVID-19 vaccination to VN remain unclear. It is possible that the activation of the immune system triggered by the vaccine may lead to an inflammatory response that affects the vestibular nerve. Another hypothesis suggests that viral reactivation, particularly HSV or VZV, may contribute to the development of VN following vaccination. A meta-analysis by Shafiee et al. found that the rate of VZV reactivation among individuals who received the COVID-19 vaccine was 14 per 1000 vaccinations (95% CI 2.97–32.80). Additionally, their meta-analysis showed that HSV reactivation occurred at a rate of 16 per 1000 vaccinations (95% CI 1.06–46.4)^16^. Viral reactivation tends to occur more often in older adults due to weakened cell-mediated immunity, a process referred to as immunosenescence^17^. Further research is needed to elucidate the precise mechanisms and identify potential risk factors for VN post-vaccination. To date, there have been no reported cases of bilateral vestibulopathy in the literature, even though it is anticipated to be the most common manifestation of a systemic autoimmune condition. 

The findings of this systematic review have several implications for healthcare professionals and public health authorities. First, healthcare providers should be aware of the potential association between COVID-19 vaccination and VN and be prepared to recognize and manage such cases. Prompt diagnosis and appropriate management are crucial to ensure optimal outcomes for affected individuals. Second, public health authorities should continue to closely monitor the safety profile of COVID-19 vaccines and actively collect data on adverse events. Timely reporting and analysis of these events can contribute to a better understanding of their incidence, risk factors, and potential preventive measures. 

Moving forward, future research should focus on conducting larger population-based studies to determine the true incidence of VN following COVID-19 vaccination. These studies should employ standardized diagnostic criteria and rigorous methodologies to ensure the reliability and comparability of the data. Long-term follow-up of vaccinated individuals can provide valuable insights into the natural course and outcomes of VN post-vaccination. Additionally, investigations into the underlying mechanisms of VN following COVID-19 vaccination are warranted. Studies exploring the immunological response and inflammatory markers in affected individuals can shed light on the pathophysiology of vaccine-related VN. Furthermore, research should aim to identify potential risk factors, such as individual characteristics or vaccine-related factors, that may increase the susceptibility to VN post-vaccination. 

One of the main limitations of this review article is the potential for selection bias due to the reliance on published case reports. This may result in an overrepresentation of severe or unique cases, potentially skewing the understanding of the overall incidence and prevalence of vestibular neuritis following COVID-19 vaccination. Reporting bias is also a concern, as positive findings are more likely to be published, leading to an overestimation of the association. The lack of a control group limits the ability to establish a causal relationship, while the heterogeneity of data from various sources makes generalization challenging. These limitations emphasize the need for more comprehensive studies with larger sample sizes and rigorous designs to accurately assess the relationship between COVID-19 vaccination and VN.

## Conclusion

In conclusion, this systematic review suggests a potential association between COVID-19 vaccination and the occurrence of vestibular neuritis based on reported cases. Although rare, healthcare providers and public health authorities should remain vigilant and proactive in monitoring and addressing adverse events. Continued surveillance, further research, and collaboration among researchers, healthcare professionals, and regulatory agencies are essential to ensure the ongoing safety and efficacy of COVID-19 vaccines.
